# Engineered Bacterial Cellulose Nanostructured Matrix for Incubation and Release of Drug-Loaded Oil in Water Nanoemulsion

**DOI:** 10.3389/fbioe.2022.851893

**Published:** 2022-03-09

**Authors:** Concetta Di Natale, Vincenza De Gregorio, Elena Lagreca, Francesca Mauro, Brunella Corrado, Raffaele Vecchione, Paolo Antonio Netti

**Affiliations:** ^1^ Interdisciplinary Research Centre on Biomaterials, University of Naples Federico II, Naples, Italy; ^2^ Istituto Italiano di Tecnologia, Naples, Italy; ^3^ Department of Chemical Materials, Industrial Production Engineering, University of Naples Federico II, Naples, Italy

**Keywords:** bacterial cellulose, drug delivery, nanocellulose network, nanoemulsion, antioxidant

## Abstract

Bacterial cellulose (BC) is a highly pure form of cellulose produced by bacteria, which possesses numerous advantages such as good mechanical properties, high chemical flexibility, and the ability to assemble in nanostructures. Thanks to these features, it achieved a key role in the biomedical field and in drug delivery applications. BC showed its ability to modulate the release of several drugs and biomolecules to the skin, thus improving their clinical outcomes. This work displays the loading of a 3D BC nanonetwork with an innovative drug delivery nanoemulsion system. BC was optimized by static culture of SCOBY (symbiotic colony of bacteria and yeast) and characterized by morphological and ultrastructural analyses, which indicate a cellulose fiber diameter range of 30–50 nm. BC layers were then incubated at different time points with a nanocarrier based on a secondary nanoemulsion (SNE) previously loaded with a well-known antioxidant and anti-inflammatory agent, namely, coenzyme-Q10 (Co-Q10). Incubation of Co-Q10–SNE in the BC nanonetwork and its release were analyzed by fluorescence spectroscopy.

## 1 Introduction

In the last few years, the choice of appropriate drug delivery systems has achieved great attention in the pharmaceutical field. A successful drug delivery is influenced by several factors (La Manna et al., 2021b; Di Natale et al., 2021d) including the identification of a suitable biomaterial (Lagreca et al., 2020) to be used as a building block for the assembly of the final system (Del Valle et al., 2009). For example, very recently, nanostructure plasmalogen-loaded cubosomes or hexosomes were reported as innovative delivery systems for the lipophilic antioxidant compound, opening new opportunities for bioinspired nanoassemblies (Angelova et al., 2021; Mathews et al., 2021). In this context, another interesting material, which is synthesized from bacteria and presents a nanostructured matrix useful for drug encapsulation and release, is the bacterial cellulose (BC); it possesses a great versatility in terms of *in situ* modulation, post-synthesis chemical modifications, biocompatibility, or ease of sterilization (Barud et al., 2016; Carvalho et al., 2019). In addition, it also shows high purity and water absorption capacity, as well as single mechanical properties, good permeability, or resistance to degradation (Badshah et al., 2020; Parte et al., 2020; Gregory et al., 2021). Thanks to these properties, BC is achieving great interest in biomedical research concerning, for example, the wound dressing for skin burns or the microsurgery for the restoration of artificial blood vessels (Klemm et al., 2001; Carvalho et al., 2019). From the chemical point of view, BC is organized in a tridimensional (3D) nanofibrillar network, and this singular property makes it a suitable macromolecular support for drug encapsulation and, therefore, for the development of specific controlled release systems (Tan et al., 2019). Several studies displayed the ability of BC networks to modulate the release and bioavailability of drugs in percutaneous administration, and hence, they were suggested as supports for topical or transdermal drug delivery (Almeida et al., 2014). For example, BC fibers loaded with silver nanoparticles, in topic formulations, demonstrated an antibacterial activity up to 99.99% against *Escherichia coli* and *Staphylococcus aureus* (Fortunati et al., 2014; Volova et al., 2018). Other studies instead showed the ability of nanofibrils as aerogels to encapsulate drugs such as anti-inflammatories, anticancer, steroids or biomolecules as peptides, and proteins or antibodies (Gopi et al., 2018). Apart from drug loading, the possibility of regulating their release is also remarkable, and BC nanofibers revealed the ability to modulate the release of both hydrophobic and hydrophilic compounds, thus providing versatile materials with respect to drug delivery (Picheth et al., 2017). Several studies, indeed, revealed as BC nanofibers can allow a sustained and controlled release of antioxidant molecules such as quercetin and vanillic or cinammic acid for food or cosmetic applications (Trombino et al., 2008; Li et al., 2019; Morais et al., 2019).

The present work proposes a method to load the BC nanonetwork with stabilized lipophilic compounds and allow its release in a time frame of hours which is compliant with skin applications. To the best of our knowledge, no Co-Q10 nanocarrier has ever been encapsulated within cellulose fibers. In detail, we used an ultra-stable oil-in-water (O/W) SNE coated by a thin layer of chitosan (CT) (Vecchione et al., 2016a; Vecchione et al., 2016b; Vecchione et al., 2014), a positively charged polyelectrolyte, able to encapsulate lipophilic molecules such as curcumin (Fotticchia et al., 2017; Vecchione et al., 2017; Langella et al., 2018; Vecchioneet al. 2016), lycopene (Quagliariello et al., 2018), and Co-Q10 (Quagliariello et al., 2020). In the latter stage of work, the nanocarriers were loaded with Co-Q10 acting as antioxidant and anti-inflammatory agents meant for oral delivery, proving high loading capability and molecular stability preservation. However, Co-Q10 is also very well-known as an antioxidant for skin applications (El-Leithy et al., 2017). Starting from these considerations, here, we propose the development of a BC-Co-Q10-SNE nanonetwork for a double release approach where the cellulose network releases the Co-Q10–loaded SNE which, upon degradation, can finally release active Co-Q10 to the skin. Our BC was produced by the SCOBY using optimized conditions in terms of humidity (98%) and temperature (30°C), as well as the culture media volume that assured the correct moist status, avoiding the production of a thick layer with BC exfoliation (Alkhalifawi and Hassan, 2014; Lahiri et al., 2021). Several spectroscopic techniques such as scanning electron microscopy (SEM) and infrared ray (IR) were used for BC morphological and chemical characterizations, while Co-Q10-SNE loading and release were studied by confocal microscopy and fluorescence, respectively, by following Co-Q10 autofluorescence.

This study aims to be a proof of concept for a new use of BC as a drug delivery system; future analysis will indeed focus on the production of inflamed micro-tissues which will subsequently be treated with the BC-Co-Q10-SNE described in this study.

## 2 Material and Methods

### 2.1 Materials

Both soybean oil (density at 20°C of 0.922 g ml^−1^) and the surfactant Lipoid E80 (egg lecithin powder 80–85% enriched with phosphatidyl choline (PC) and 7–9.5% content in phosphatidyl ethanolamine (PE)) were purchased from Lipoid GmbH and used without further purification. Millipore Milli-Q water was used for the preparation of all nanoemulsions and solutions. Chitosan (CT, LMW 90–150 kDa, and DDA 84% determined *via* 1H-NMR) was purchased from Sigma-Aldrich (Milan, Italy). Ubidecarenone, coenzyme-Q10 (Co-Q10), was kindly offered by the Faravelli Group. Kombucha SCOBY was purchased from KEFIRA, and glucose and agar were purchased from Sigma-Aldrich.

### 2.2 Methods

#### 2.2.1 Media Preparation and SCOBY Culture

For Kombucha SCOBY (KEFIRA) culture, a tea broth and an agar plate were prepared with the following protocol: 860 ml of deionized water (dH_2_O) was boiled before adding 140 g/L of glucose; 10 sachets (20 g) of black tea were added and steeped for 10 min. Consequently, the tea bags were removed, and the sweetened tea was cooled at room temperature; then, apple vinegar (140 ml/L) was added. The medium was autoclaved at 121°C for 15 min. For solid medium, the agar was autoclaved separately. To improve the fermentation process of the SCOBY, one piece (1 × 1 cm) of the SCOBY was aseptically added into the liquid broth and cultured for 3 days. Then, an aliquot of 1 ml of the previously fermented SCOBY, which acts as a starter, was inoculated into the culture broth at a concentration of 0.05% (1 ml/20 ml). For BC production, starters of the SCOBY were cultured in 50 ml tubes, and two different experimental phases were performed: uncontrolled fermentation conditions (UCC) and controlled fermentation conditions (CC). For the CC, the static fermentation process took place in a dark CO_2_ incubator in a controlled humidified atmosphere (≥80%) with constant temperature at 30°C for 3 days, in order to guarantee an optimal environment for symbiont growth. The cap of the tube was removed and perforated parafilm, previously sterilized, and was used to cover the lid and increase the exchange of O_2_ with the external surface. This process was repeated in triplicates. Tests were performed in triplicates as well. Viable count assay was performed, as reported earlier.

#### 2.2.2 Live/Dead Assay

In order to select the SCOBY pieces to use for the experimental phase, the viability percentage was assessed by using the Live/Dead BacLight Bacterial Viability Stain Kit (Molecular Probes, Eugene, and OR). First, the best concentration of the viability kit stain mixture (SYTO9 and propidium iodide (PI)) was selected, which allowed us to distinguish live cells from dead cells (SYTO9: PI, 1:2 v/v). Briefly, freshly grown SCOBY pieces were opportunely cut, harvested, and washed three times with 0.85% sodium chloride (NaCl) solution. Then, 30 μl of a mixture of SYTO9 and PI (1:2) was diluted in a final volume of 5 ml, and each SCOBY piece was incubated with 1 ml in darkness for 15 min at room temperature, according to the manufacturer’s instructions. The non-viable SCOBY were prepared by 95% ethanol treatment of bacteria for 30 min (positive control). The SCOBY pieces were washed twice with 0.85% NaCl after the treatments and examined under a confocal microscope (Confocal Leica TCS SP5 II femtosecond laser scanning system, Leica). Filters were set to 493–522 nm for SYTO9 and 618–676 nm for PI. Confocal images were obtained with 40x objective (optical zoom 1.5). Each sample was scanned at randomly selected areas as a series of vertical optical sections, each one 0.50 μm thick. Quantitative analyses of each SCOBY piece were carried out by analyzing the digital images of live (green) and dead (red) bacteria by ImageJ software. Each image was divided in regions of interest (ROIs) with comparable areas, and thresholding was performed. The fluorescence intensity per unit area was measured and calculated as the percentage of viable cells. Thereafter, the culture parameter was set, maintaining the temperature at 30°C and the humidity (>98%) for controlled experimental cultures (CCs). To obtain the CC condition, the samples were placed in an incubator at 30°C by inserting a 10-cm high tank with an evaporating surface of 20 cm to obtain constant humidity >80% without water refill during the entire experimental phase. Conversely, for uncontrolled experimental cultures (UCCs), SCOBY pieces were cultured at room temperature (∼23°C) and environmental humidity (∼50%).

#### 2.2.3 Ultrastructural Characterization of the BC Layer

For ultrastructural analysis of the fibrillar structure of BC, the BC layers obtained by UCC and CC were primarily fixed with 4% paraformaldehyde. Then, it was fixed with 2.5% of glutaraldehyde in 0.1 M of sodium cacodylate and was left for 1–4 h at the room temperature. In due course of time, it was washed thrice for 10 min in 0.1 M sodium cacodylate and sucrose and buffered at the normal temperature. It was then buffered with 1% osmium tetroxide (OsO_4)_ in 0.1 M sodium cacodylate for 1 h at 4°C and afterward again, washed thrice with 0.1 M sucrose buffer solution. Dehydration was performed on the sample using ethanol at 30, 50, 70, and 95% for 15–60 min at 4°C. Finally, 100% of ethanol was applied for 15–60 min at the room temperature thrice. Image analyses were performed by ImageJ software by using the DiameterJ plugin (Hotaling et al., 2015). First of all, the scale bar of the image was measured by using the scale option after using the zoom option from the toolbar. SEM images (1024 × 768 pixels) were obtained, as already described (Di Natale et al., 2019; Bagheri et al., 2021; Di Natale et al., 2021a; Di Natale et al., 2021b; Di Natale et al., 2021c; Di Natale et al., 2021d; Florio et al., 2021; Di Natale et al., 2020a; Di Natale et al., 2020b; La Manna et al., 2021a; La Manna et al., 2021b), and then segmented using the algorithms provided by “DiameterJ Segment” to convert the image into binary forms. Then, segmented images were processed by DiameterJ to measure the diameter of the cellulose bundles and fibers. In addition, DiameterJ was also used to measure the BC network parameters as the mean pore area, porosity percent, and numbers of pores:

$$Mean\;pore\;area=\frac{\text{TOTAL}\;\text{NUMBER}\;\text{OF}\;\text{BLACK}\;\text{PIXEL}\;\text{COUNTED}\;\text{IN}\;\text{PORES}}{\text{TOTAL}\;\text{NUMBER}\;\text{OF}\;\text{PORES}\;\text{IN}\;\text{IMAGE}},$$ (1)

$$\%\;porosity=\frac{\text{TOTAL}\;\text{NUMBER}\;\text{OF}\;\text{BLACK}\;\text{PIXELS}}{\text{TOTAL}\;\text{NUMBER}\;\text{OF}\;\text{PIXELS}\;\text{IN}\;\text{IMAGE}}.$$ (2)

#### 2.2.4 Infrared Spectroscopy

The BC chemical structure was confirmed by IR. BC sheet of 1 cm. The measurements were carried out in the range of 500–4,000 cm^−1^ in absorption or transmission modes (64 scans, 4 cm^−1^ resolution) (Thermo Fisher Scientific Instruments, Nicolet 6,700, Waltham, MA, United States). The spectra were subject to ATR correction, smoothing, and baseline correction to be normalized (Di Natale et al., 2021a).

#### 2.2.5 Co-Q10-SNE Production and Characterization

At first, a primary Co-Q10 negatively charged oil-in-water (O/W) nanoemulsion (NE) at 20 wt% of oil concentration was prepared, as previously reported (Vecchione et al., 2014; Quagliariello et al., 2020; Profeta et al., 2021). Briefly, first the oil phase was obtained by adding the surfactant to the soybean oil. For the analysis, 5.8 g of Lipoid E80 was dissolved in 24 ml of soybean oil at 60°C and mixed using the immersion sonicator (Ultrasonic Processor VCX500 Sonic and Materials). An amount of 4.08 g of Co-Q10 was dissolved in the oil phase at 60°C for 1 h, then added dropwise to the aqueous phase (Milli-Q water), and mixed again using the immersion sonicator. The pre-emulsion was passed at 2000 bar through the high-pressure valve homogenizer (Microfluidics M110PS) for three individual cycles to greatly reduce the initial size; then, the reservoir was continuously refilled for 200 steps.

Co-Q10-NE was then functionalized with CT to have a positively charged SNE. In detail, to achieve the secondary emulsion, a first layer of CT was deposited above the primary one by following an already developed procedure (Vecchione et al., 2014; Vecchione et al., 2016). Briefly, a 0.1 M acetic acid solution of CT pH 4 (0.2 wt%) was prepared, and the 20 wt% oil-O/W NE was then added to the CT solution under vigorous stirring for 15 min to allow uniform CT deposition. Final concentrations of oil and CT were 10 and 0.1 wt%, respectively, while the pH of the final NE (SNE) was 4. O/W NE and SNE were characterized by measuring the size, polydispersity index (PDI), and ζ-potential values through a dynamic light scattering (DLS) instrument (Zetasizer ZS, Nanoseries ZEN 3600, Malvern Instruments Ltd., Malvern, United Kingdom, λ = 632.8 nm). All the samples were diluted up to a droplet concentration of approximately 0.025 wt% by using Milli-Q water. A detecting angle of 173 was used. A default refractive index ratio (1.5900) and three runs for each measurement (1 run lasting 100 s) were used in the calculations of the particle size distribution. ζ-potential analysis was carried out by setting 30 runs for each measurement. The morphology of Co-Q10-SNE was observed by Cryo-TEM analysis. For the preparation of the frozen-hydrated sample, the plunge freezing method was performed. Briefly, a drop of 3 μl of the sample was deposited on 200-mesh holey carbon grids (Ted Pella, United States); then, it was inserted in the chamber of a FEI Vitrobot Mark IV (FEI Company, the Netherland) at 4°C and 90% of humidity. The droplet of the sample was blotted with a filter paper for 1 s (blot force 1, drain time 0.5 s) and then, the grid was plunged into liquid propane. Then, the grid was stored in liquid nitrogen in a grid box until it was finally transferred to a cryo-specimen 626 holder (Gatan, Inc., United States) and loaded into the Cryo-transmission electron microscope for imaging. To obtain the image of the nanocarriers, we used a Tecnai G2 20, a Cryo-TEM transmission electron microscope (FEI Company, the Netherlands) equipped with a LaB6 emitter (acceleration voltage of 200 kV), and recorded with a 2 × 2 k CCD-Eagle 2HS camera. The frozen-hydrated sample is a radiation-sensitive material, so to avoid damaging it, the observation was carried out in a low-dose mode.

#### 2.2.6 Co-Q10-SNE Encapsulation and Release: Confocal Microscopy and Fluorescence

For the experiment, 5 mg of cellulose layers (5 mm diameter) were suspended in 1.5 ml of Co-Q10-SNE and incubated at room temperature at different time points (15 min and 30 min) with a gentle agitation. All the tests were executed in triplicates. All samples were stored at 4°C, and SNE adsorption on cellulose layers was evaluated by confocal microscopy. Samples were imaged using a Leica TCS SP5 STED-CW gated microscope (Leica-Microsystems, Mannheim, Germany) with HCX IRAPO L 25.0 × 0.95 water objective (Di Natale et al., 2020a; Jamaledin et al., 2020; Di Natale et al., 2021a; Battisti et al., 2019; La Manna et al., 2021a). A laser source of 488 nm was used to excite the Co-Q10 in the oil core. Moreover, a semi-quantitative analysis was performed on at least five images for each z-plane to obtain the mean fluorescence intensity of the loaded Co-Q10-SNE. Using ImageJ software, the mean gray value (MGV) of the green channel was measured for each image (Celetti et al., 2016; Di Natale et al., 2018). Co-Q10-SNE release studies were carried out by suspending 5 mg of BC-Co-Q10-SNE in 1.5 ml of water. Samples were incubated at 37°C and shaken under gentle conditions. At fixed time points (15, 30 min, 1, 2, 3, and 24 h), 1 ml of the sample was withdrawn after cellulose layer sedimentation using centrifugation for 5 min at 10,000 rpm (MicroCL21R, Centrifuge, Thermoscientific, United States). The pellet was resuspended in the same volume of fresh buffer. The collected supernatants were analyzed by fluorescence (Microplate Readers Perkin Elmer); the excitation wavelength was 450 nm, and the maximum emission was recorded between 470 and 600 nm. The fluorescence intensity peak was determined at 551 nm. All the tests were executed in triplicates.

## 3 Results and Discussion

### 3.1 Cellulose Production: Morphological and Chemical Characterization

**FIGURE 1:**
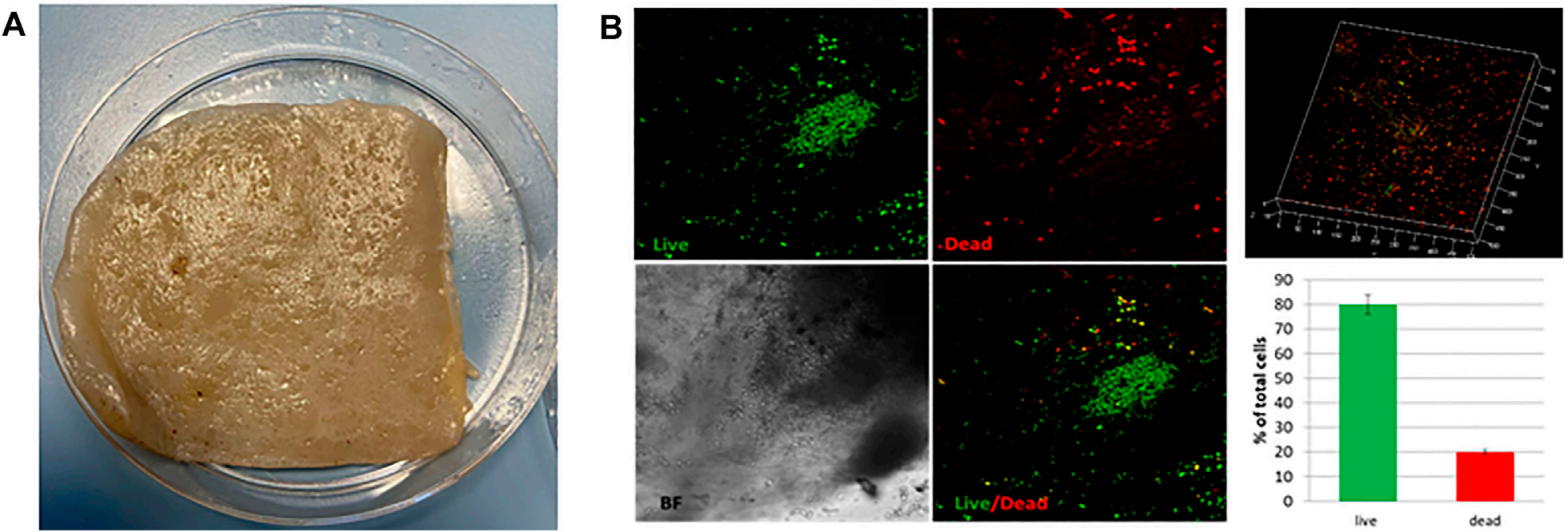
**(A)** Representative image of the SCOBY portion for BC production. **(B)** Live/Dead fluorescence images of the SCOBY. 3D reconstruction of the observed portion and the percentage of live (green) and dead (red) bacteria.

**FIGURE 2:**
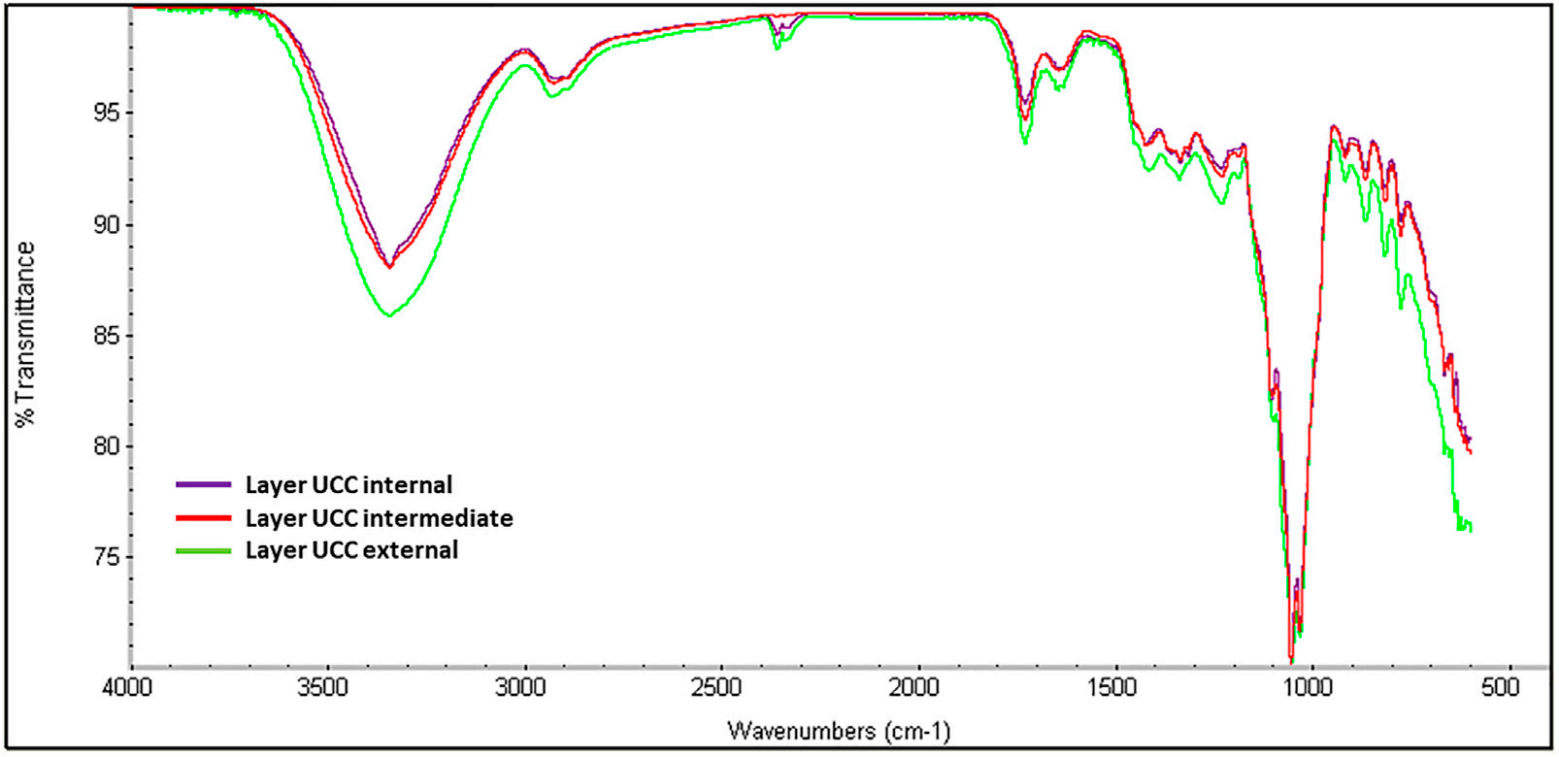
IR analysis of UCC internal, intermediate, and external layers obtained at 72 h.

**FIGURE 3:**
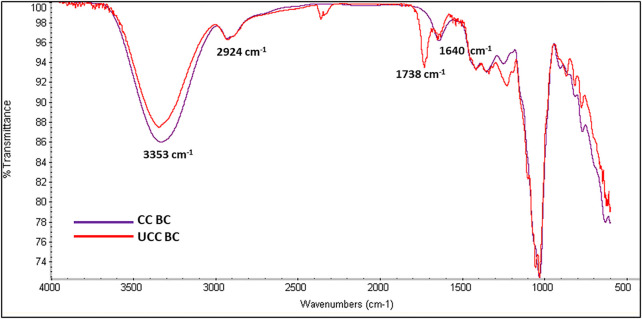
IR spectra of CC (violet spectrum) and UCC BC (red spectrum) at 72 h.

In order to validate the SCOBY **(** Figure 1A **)** for BC production in static culture, preliminary viability assay was performed by using Live/Dead assay. SCOBY portions (1cmx1cm) were observed under a confocal microscope in the central and peripheral areas, obtaining a reliable measurement of the entire sample. SCOBY pieces on which symbiont viability reaches at least 50% were used for the experimental phase, as reported in representative images and 3D reconstruction (Figure 1B). Quantitative analysis of the SCOBY portion showed a viability of 79 ± 3.5% by measuring the intensity of green (for viable bacteria) and red (for non-viable bacteria) fluorescence measured by the area. SCOBY pieces with a strong reduction in the cell viability were not used for the experimental phase (data not showed). Once the SCOBY pieces to use for BC production are selected, two different culture conditions were set: a temperature of 30°C and the humidity >98% for the controlled experimental culture (CC) and room temperature (∼23°C) and environmental humidity (∼50%) for the uncontrolled experimental culture (UCC). To carry out the morphological characterization of the BC produced in UCC at different stages of maturation, BC layers were produced in static conditions without refreshing the medium. Each layer (about 1 mm thick) was separated from the layer below due to a variation of the medium/air interface and a reduction in the volume of the medium over time with a reduced humidity. The BC layers obtained with this procedure showed different ripeness degrees, starting from the bottom (in direct contact with the liquid suspension) with the lowest ripeness up to the more superficial ones (in direct contact with air), which appear to be, from a macroscopic analysis, more consistent and thicker >1 mm. These tests made it possible to observe a variation in the cellulose consistency based on the degree of maturation whose chemistry was studied by IR spectroscopy. In detail, three layers (internal, intermediate, and external) were obtained at 72 h, and they showed the typical BC peaks (Keshk et al., 2009; Castro et al., 2011; Tabarsa et al., 2017) with the bands at 3,353 cm^−1^ and 2924 cm^−1^ relative to the stretching of the OH and CH groups, respectively; a peak observed at 1738 cm^−1^ and 1,640 cm^−1^ associated with the stretching of the C = O groups, and the bending of the OH groups referred to absorbed water molecules into cellulose fibers, a peak at 1,046 cm^−1^ corresponding to the vibration of the pyranose ring–C–O–C, and the peak at 889 cm^−1^ related to the presence of β-glycosidic bonds (Figure 2). The three layers also revealed a similar degree of polymerization with the presence of the peak at 1738 cm^−1^, even if, its intensity grows as the superficiality of the layer increases (Figure 2). To avoid the development of this crosslinked BC, which could hinder the correct SNE incubation, BC was grown under CC conditions, as explained earlier, and the correct maturation level was analyzed by IR. From the morphological analysis, the samples grown under CC conditions appear to have an adequate hydration status, highlighted by the lower degree of compactness, as well as greater transparency. In contrast, the unique layer produced under UCC conditions is thicker and not transparent at all, indicating a greater degree of compactness and less hydration, which is reflected in a greater degree of crosslinking in the IR spectrum (Figure 3 red spectrum**)**. Spectra of UCC and CC BC obtained at 72 h, corroborated our hypothesis; indeed, BC obtained in CC conditions showed only the maturation peak at 1,640 cm^−1^ (Figure 3, violet spectrum) in contrast with the UCC BC which revealed the crosslinked band at 1738 cm^−1^ (Figure 3 red spectrum). IR spectra strongly confirmed that the moisture content seemed to be directly related to the degree of compactness (revealed by the crosslinking) of BC, demonstrating that the adjusted humidity environment allows reaching a loosening of the cellulose structure useful for nanocarriers’ penetration upon incubation.

### 3.2 Ultrastructural Characterization of BC

**FIGURE 4:**
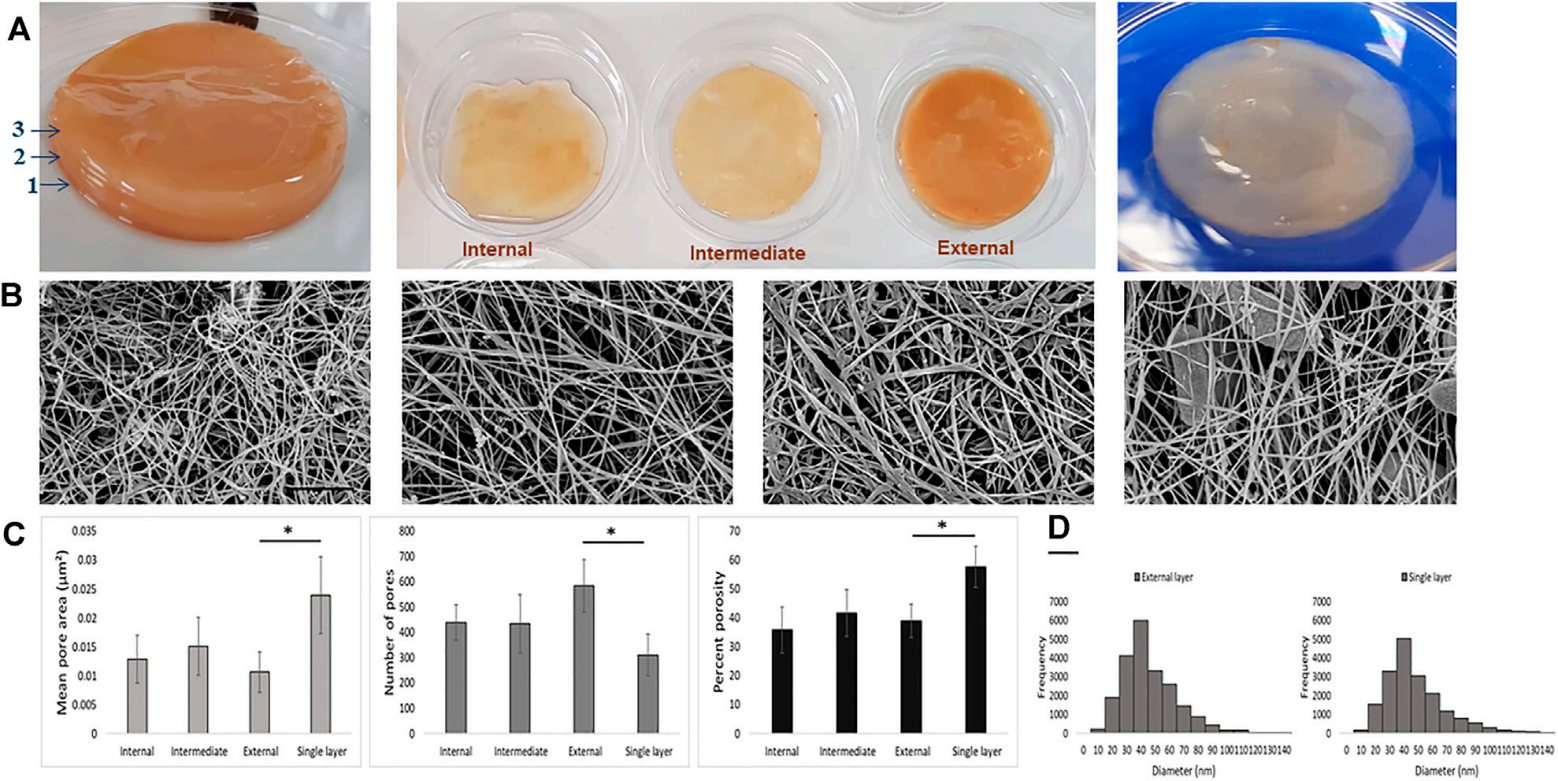
**(A)** Qualitative images of BC layers produced under UCC (left and middle) and CC (right). **(B)** Representative SEM images of the different BC layers (internal, intermediate, external, and single layer, from left); scale bar 1 µm. **(C)** Mean pore area, number of pores, and porosity percentage of BC networks of the different BC layers. **(D)** Fiber diameter histogram of external and single BC layers produced in UCC and CC, respectively.

To achieve the ultrastructural characterization of the BC produced in UCC or in CC, BC layers were produced in static conditions without refreshing the medium (Figure 4A). Representative SEM images showed a different degree of compactness of the cellulose fiber network. In particular, the external layer in UCC highlighted a greater degree of compactness, as shown in Figure 4B. Quantitative data of the SEM images showed for the single layer obtained in CC a significantly greater mean pore area and porosity percentage with a smaller number of pores (*p* < 0.05) than the layers obtained in UCC, especially the superficial layer (External). In detail, the porosity values are 39.0, 41.7, and 35.9, while the mean pore area values are 0.0107, 0.0151, and 0.0129 µm^2^ for the external, intermediate, and internal layers, respectively, indicating a slight difference between the superficial layer and the most internal layers obtained in UCC although not statistically significant (Figure 4C).

At last, all samples showed the fiber diameters in a range of 30–50 nm, and a comparable fiber frequency was also reported (Figure 4D), indicating that the main difference among BC produced by the SCOBY is in assembly of the fiber rather than in the single fibers.

### 3.3 Co-Q10-SNE Encapsulation and Release by BC

As stated in the Materials and Method section, primary and secondary Co-Q10-NEs were produced by a method developed in our laboratory (Quagliariello et al., 2020). Co-Q10-NE and Co-Q10-SNE size distribution and uniformity were evaluated by DLS measurements, as reported in Supplementary Figures S1A, B. In detail, Co-Q10-NE displayed an average size of 112.4 ± 0.65 nm with a PDI of 0.12 ± 0.04 and a surface charge of −46.8 ± 0.40 mV (Supplementary Figure S1C), while Co-Q10-SNE reported an average size of 103.0 ± 1.0 nm with a PDI of 0.090 ± 0.025 and a charge of -+39.9 ± 0.07 mV (Supplementary Figure S1C), in agreement with those reported in the literature (Vecchione et al., 2016; Quagliariello et al., 2018; Vecchione et al., 2014; Profeta et al., 2021). Before starting incubation experiments, we also evaluated the chemical stability of our SNEs over time, and as shown in Supplementary Figure S1D, they remained stable for up to 30 days, without any variation in size or PDI. Nanocarriers’ uniformity and stability were also confirmed by Cryo-TEM analysis (Supplementary Figure S2), where Co-Q10-SNE showed monodispersed spherical nanostructures of ∼100 nm.

**FIGURE 5:**
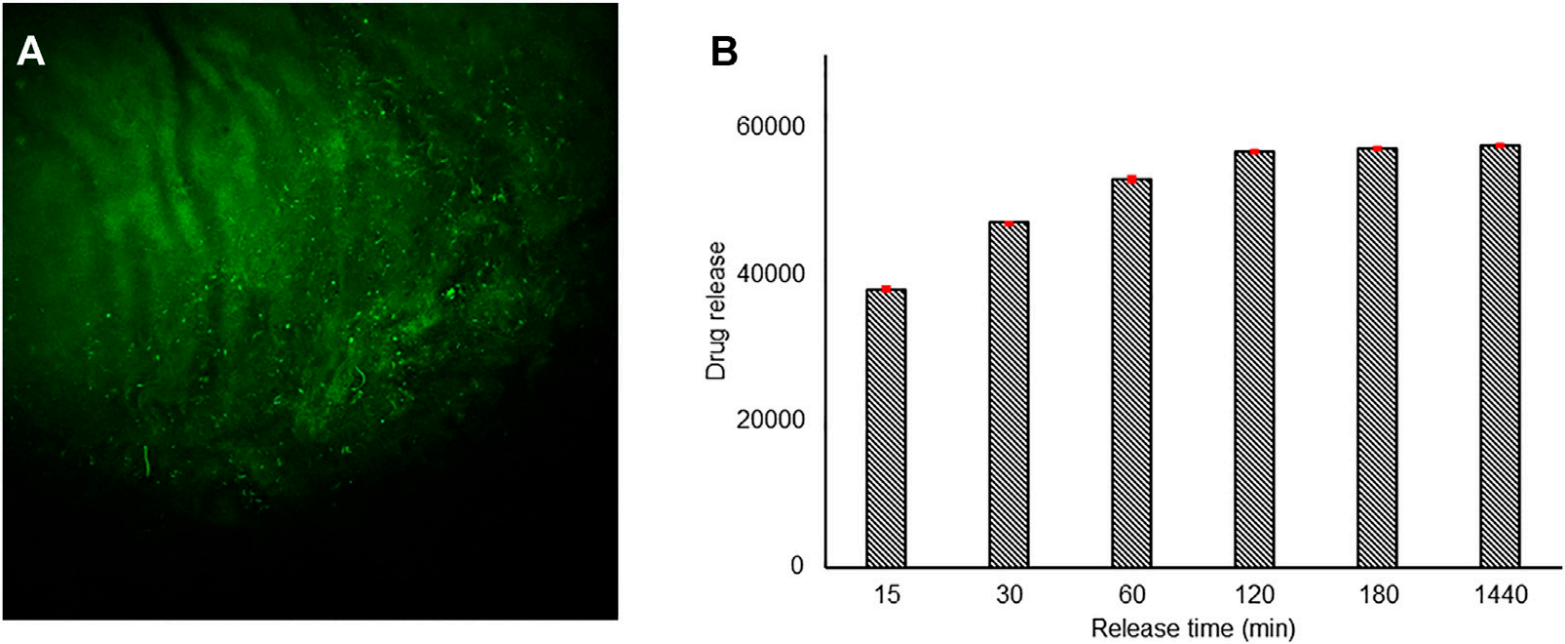
Confocal images of the BC-Co-Q10 SNE, **(A)** 15 min incubation time λ_exc_ 450 nm, λ_emiss_ 470–600 nm. **(B)** Release kinetic studies of the Co-Q10 SNE from BC incubated for 15 min.

As to incubation experiments, 1cm x 1cm layers of BC were used and incubated at two different time points (15 and 30 min) with 1 ml of Co-Q10-SNE. The correct incubation was then evaluated by confocal microscopy, following the autofluorescence of Co-Q10 at 551 nm, as shown in Figures 5A,B and Supplementary Figure S3. Confocal analysis revealed no difference in the fluorescence intensity of both samples **(** Figure 5A, Supplementary Figure S3A **)** underlying as the ultrastructure of BC allows a complete loading of SNEs already at 15 min. This result was corroborated by Co-Q10-SNE release studies where similar quantities of the Co-Q10-SNE were released from both BC samples. These analyses were carried out by fluorescence, following the maximum of Co-Q10-SNE emission at 551 nm. In detail, 5 mg of BC-Co-Q10-SNEs were suspended in 1.5 ml of water and incubated at 37°C for different time periods from 15 to 1,440 min and after 1 ml of the supernatant was removed at each time and analyzed. The quantification of release kinetics showed that for both samples (BC incubated at 15 and 30 min), the Co-Q10-SNE fluorescence signal increased during the time reaching the saturation point from 120 to 1.440 min (Figure 5B, Supplementary Figure S3B). These results were reached optimizing the BC preparation process to obtain a single not a crosslinked layer of nanofibers. Indeed, by conducting preliminary experiments on UCC BC layers, we noted that more external layers were not able to incubate the Co-Q10-SNE, and encapsulation is mostly superficial in all the samples analyzed, both at 15 and 30 min **(** Supplementary Figures S4A–F).

The reported results show that the BC networks produced with our conditions can carry out a sustained release of drugs. Our double release approach based on the use of a drug-encapsulated nanocarrier within BC could help obtain a prolonged antioxidant drug release, enhancing their therapeutic effects; it is, indeed, reported that encapsulated antioxidants show a better stability, and their gradual and sustained release leads to a superior antioxidant profile (Khalil et al., 2019). However, simple drug encapsulation in BC is not able to completely achieve the described effects, for example, curcumin loaded in cellulose acetate electrospun nanofibers showed a great initial burst that gradually increased over time in an uncontrolled manner (Khoshnevisan et al., 2018). Conversely, we proposed a delivery system which can guaranty a controlled and time-sustained release highly wished for antioxidants. In addition, by tuning the SNE size (Vecchione et al., 2014), in principle, we may easily tune the release kinetics according to the required needs.

## 4 Conclusion

The current research work was carried out to evaluate the potential application of BC as a drug delivery system (an explicative final system was reported in Supplementary Figure S5). The BC layers were prepared starting from the SCOBY culture using UCC and CC conditions. Preliminary incubation studies showed that under UCC conditions, only the innermost layer, the least crosslinked layer, started to incubate the nanocarrier (Supplementary Figure S4), and therefore, to reproduce the best loading conditions, we optimized the BC preparation process, to assure the production of a mature not crosslinked BC nanonetworks. Characterization data showed that CC-BC layers were mature, and IR data corroborate these results, demonstrating the presence of chemical bands related to BC nanofibers that are not crosslinked. The Co-Q10-SNE loading and *in vitro* release studies revealed that BC matrices can encapsulate the drug already in 15 min; indeed, confocal images and fluorescence kinetic studies highlighted no differences with the BC incubated for 30 min. In detail, the quantification of release kinetics demonstrated that for both samples, the Co-Q10-SNE fluorescence signal increases in intensity during the time reaching the saturation point from 120 to 1.440 min. The obtained results concluded that our BC produced in CC conditions could represent a novel matrix for the delivery of drug-encapsulated nanocarriers. Indeed, thanks to the optimization of BC synthesis, it could guaranty enough hydration to demonstrate for the first time the ability to incubate O/W nanoemulsions, which are ideal nanocarriers for the encapsulation and stabilization of lipophilic and water-labile molecules, such as Co-Q10. Additionally, by playing with the SNE size and with BC synthesis conditions, we may modulate nanocarriers and therefore biomolecule release to the skin. However, further research works are required to explore this potential application. Future analysis will indeed focus on the production of inflamed micro-tissues that will be then healed with the BC-Co-Q10-SNE and appropriately analyzed to evaluate the therapeutic power of the proposed system.

## Data Availability

The raw data supporting the conclusion of this article will be made available by the authors, without undue reservation.
