# Supplementary material for: Engineered Bacterial Cellulose Nanostructured Matrix for Incubation and Release of Drug-Loaded Oil in Water Nanoemulsion
Source: Front Bioeng Biotechnol. 2022 Mar 9;10:851893. doi: 10.3389/fbioe.2022.851893 (PMC8959586; doi:10.3389/fbioe.2022.851893)
Supplement: Supplementary file 1 [file DataSheet1.docx]

Supplementary Material

Bacterial cellulose as novel drug-loaded nanocarrier delivery system

**Concetta Di Natale ^1,2,3^, Vincenza De Gregorio ^1,2^ Elena Lagreca ^2,3^ , Francesca Mauro ^2,3^, Brunella Corrado ^1,2^ , Raffaele Vecchione ^2*^ and Paolo Antonio Netti ^1,2,3^ .**

^1^ Interdisciplinary Research Centre on Biomaterials (CRIB), University of Naples Federico II, Naples, Italy

^2^ Istituto Italiano di Tecnologia, Largo Barsanti e Matteucci 53, Naples 80125, Italy

^3^ Department of Chemical Materials; Industrial Production Engineering, University of Naples Federico II, Naples

* Correspondence:
Raffaele Vecchione
Raffaele.vecchione@iit.it


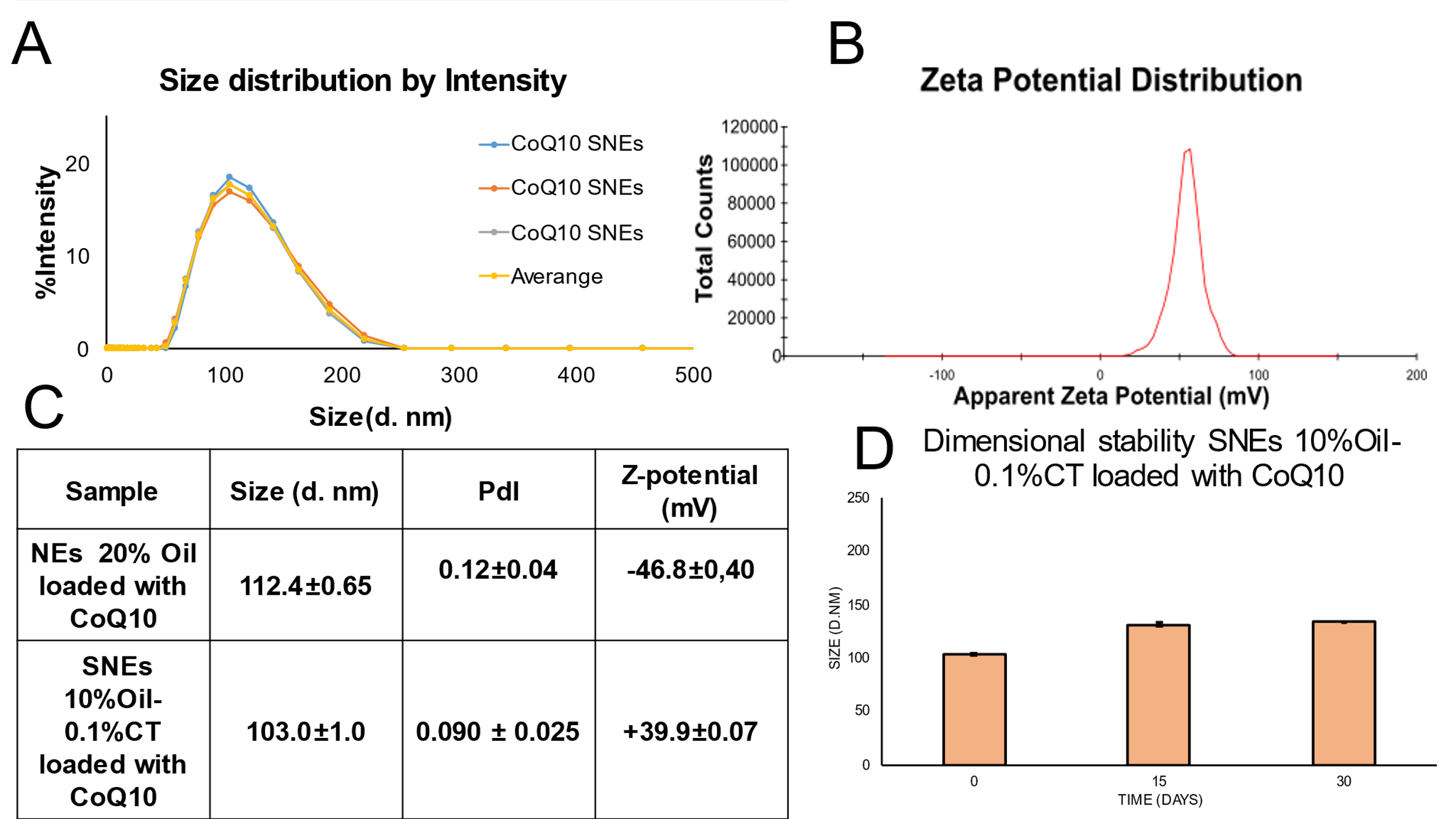


**Figure S1:** A-B) Example of Size distribution by Intensity of Co-Q10-SNE fomulations (blu- orange –gray) obtained by DLS with the average values in yellow and PDI distribution. C) Average value of size, PDI and zeta potential distribution of both Co-Q10-NE and Co-Q10-SNE. D) Dimensional Stability of Co-Q10-SNE.


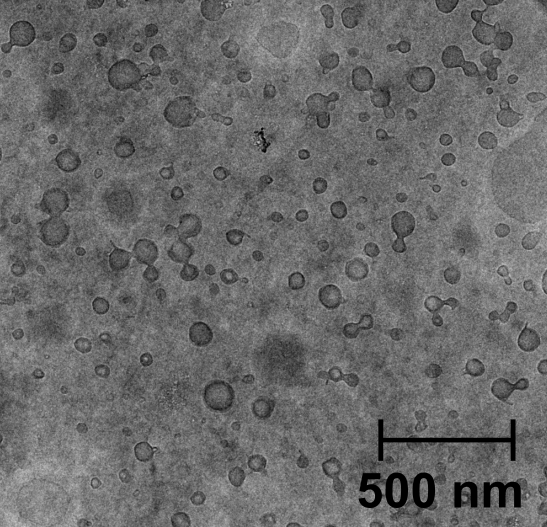


**Figure S2:** Cryo-TEM analysis of Co-Q10-SNE.


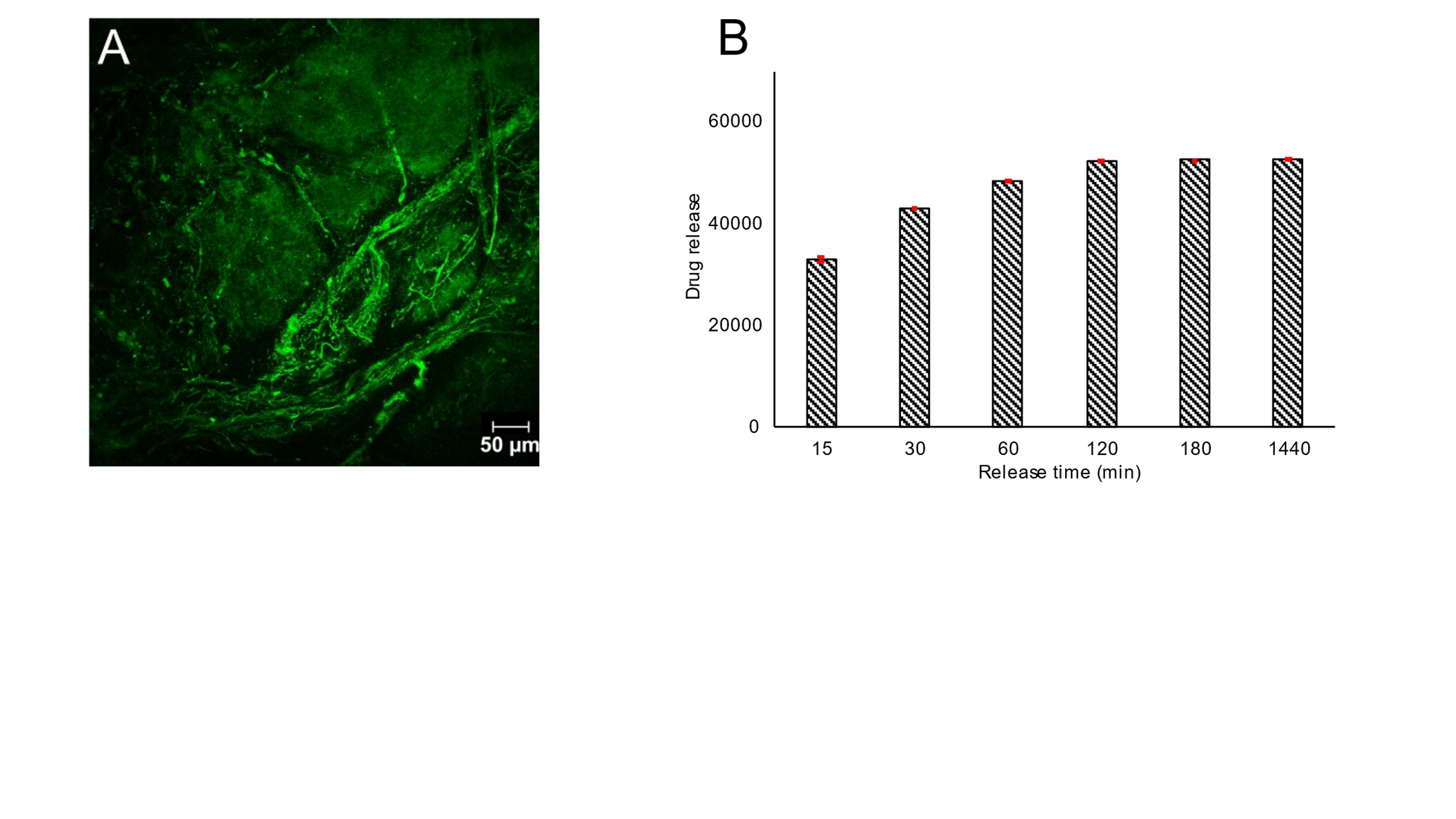


**Figure S3:** Confocal images of BC-Co-Q10-SNE, A)30 min incubation time λ_exc_ 450 nm, λ_emiss_ 470-600nm. B) Release kinetic studies of Co-Q10-SNE from BC incubated for 30 min.

**Figure S4:** Confocal images of UCC internal, intermediate, and external layer, incubated with Co-Q10-SNE at A-B-C)15 min incubation time, D-E-F) 30 min; λ_exc_ 450 nm, λ_emiss_ 470-600nm.


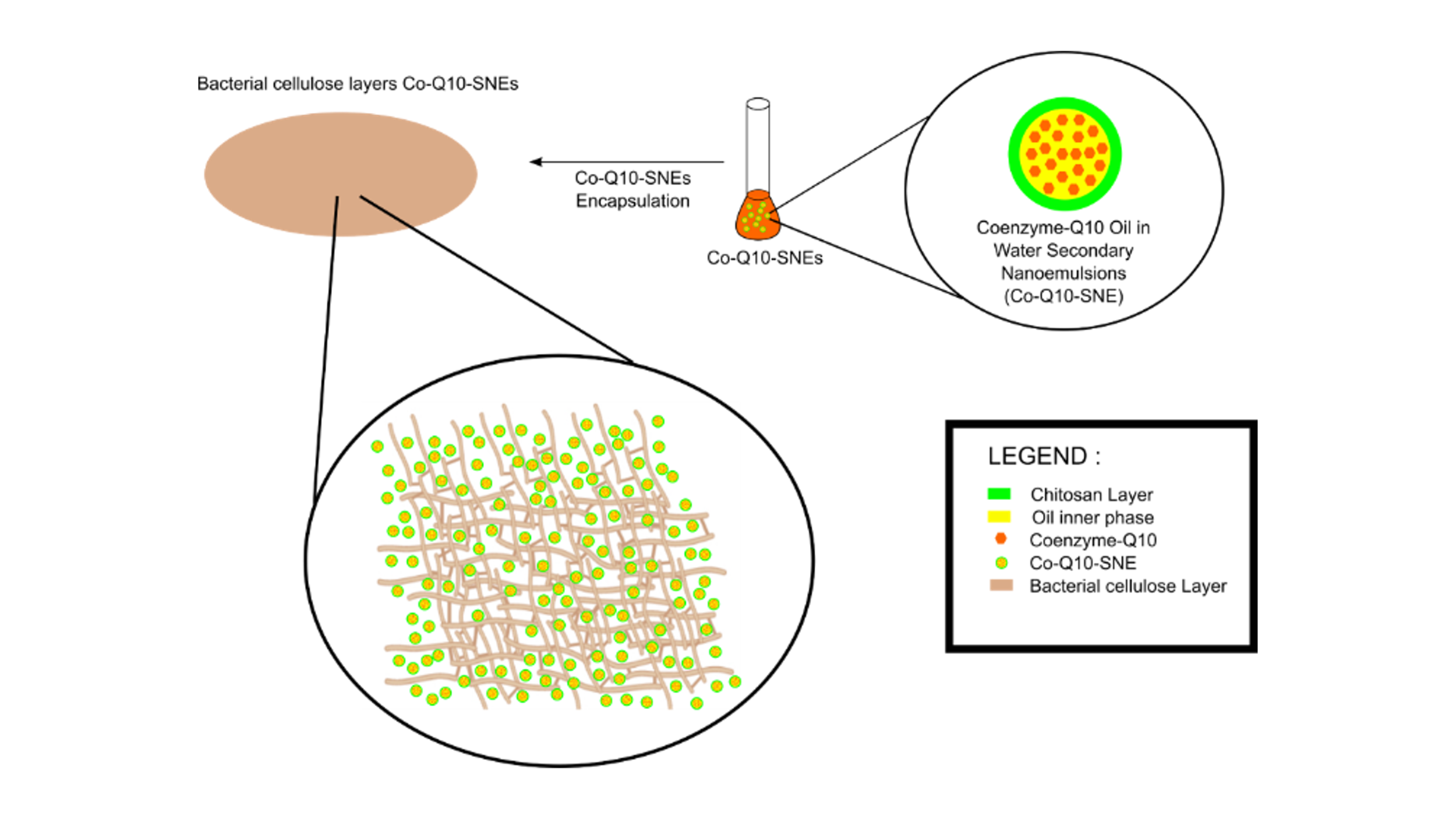


**Figure S5:** Schematic representation of BC-Co-Q10-SNE assembly.
